# 基于共价有机骨架材料的磁固相萃取-气相色谱-负化学电离源质谱法测定环境水体中3种得克隆类物质

**DOI:** 10.3724/SP.J.1123.2023.11002

**Published:** 2024-10-08

**Authors:** Yuankun LI, Chaonan HUANG, Qian ZHOU, Jiawen CHENG, Jiping MA

**Affiliations:** 1.青岛理工大学环境与市政工程学院, 山东 青岛 266525; 1. School of Environmental and Municipal Engineering, Qingdao University of Technology, Qingdao 266525, China; 2.光大理工环境技术研究院有限公司, 山东 青岛 266033; 2. Environmental Technical Research Institute of Everbright Technology Co., Ltd., Qingdao 266033, China

**Keywords:** 共价有机骨架, 磁固相萃取, 气相色谱-负化学电离源质谱, 得克隆类物质, 环境水体, covalent organic frameworks (COFs), magnetic solid-phase extraction (MSPE), gas chromatography-negative chemical ionization mass spectrometry (GC-NCI/MS), dechloranes, environmental water

## Abstract

得克隆类物质是一类添加型阻燃剂,具有持久性、毒性和生物蓄积性,可在环境介质中长期存在,并通过多种途径进入人体,对人类健康造成危害。本研究将Fe_3_O_4_磁性纳米粒子与三醛基间苯三酚-联苯胺复合材料(TpBD)相结合,制备了磁性共价有机骨架材料(Fe_3_O_4_@TpBD),并将其作为磁固相萃取吸附剂,建立了环境水体中3种得克隆类物质的气相色谱-负化学电离源质谱分析方法。利用透射电子显微镜、扫描电子显微镜、傅里叶变换红外光谱、X射线衍射等手段对Fe_3_O_4_@TpBD的形貌、粒径、表面基团和结构等进行表征,并考察了Fe_3_O_4_@TpBD用量、水样pH、萃取时间、洗脱剂种类及体积、洗脱时间和离子强度等对目标分析物萃取效率的影响。结果表明,3种目标分析物在2~1000 ng/L范围内线性关系良好,方法的检出限为0.18~0.27 ng/L,定量限为0.60~0.92 ng/L,日内和日间精密度分别为4.2%~16.2%(*n*=6)和6.9%~15.7%(*n*=6)。将该方法应用于4种实际水样中得克隆类物质的分析检测,在低、中、高3个加标水平下,3种得克隆类物质的回收率为77.8%~113.4%,相对标准偏差为2.5%~16.3%。该方法的样品前处理过程简便,灵敏度高,重复性好,适用于环境水体中得克隆类物质的分析检测。

## Abstract (en)

Dechloranes are additive-type chlorine flame retardants that are widely used in processing industrial products, such as electronic equipment and textiles. Dechloranes, which can enter the human body through various routes, pose significant health risks because of their toxicity, persistence, and bioaccumulation. In 2023, dechlorane plus was listed in the Stockholm Convention on Persistent Organic Pollutants. In the same year, China recognized this compound as a priority-controlled substance. Dechloranes are commonly found at trace levels in water, which is extremely harmful to the environment and human health. Therefore, the development of detection methods for dechloranes is crucial. Magnetic solid-phase extraction (MSPE) has attracted considerable attention because of its low organic solvent consumption, simplicity of adsorbent separation, and ease of operation. In general, the selectivity and efficiency of MSPE depend on the characteristics of the adsorbent. Covalent organic frameworks (COFs) have regular porosity, structural predictability and stability, high specific surface areas, and adjustable pore sizes, which are advantageous for a wide range of separation and analysis applications. In this study, Fe_3_O_4_ magnetic nanoparticles and a COF material (TpBD) were combined to prepare Fe_3_O_4_@TpBD as an adsorbent for dechloranes. Subsequently, an effective method for analyzing dechlorane in environmental water was established by coupling MSPE with gas chromatography-negative chemical ionization mass spectrometry (GC-NCI/MS). The successful synthesis of Fe_3_O_4_@TpBD was confirmed using transmission electron microscopy, scanning electron microscopy, Fourier transform infrared spectroscopy, X-ray diffraction, and vibrating sample magnetometry. A single-factor method was used to optimize the extraction conditions, including the Fe_3_O_4_@TpBD dosage, pH of water sample, elution solvent type and volume, extraction time, elution time, and ionic strength. The target analytes were separated on a TG-5SILMS column (30 m×0.25 mm×0.25 μm) and quantified using the external standard method in the selected-ion monitoring (SIM) mode. Under the optimal extraction conditions, the method validation results showed a linear range of 2-1000 ng/L. The limits of detection (LODs) and quantification (LOQs) were 0.18-0.27 ng/L and 0.60-0.92 ng/L, respectively, for the three analytes. The intra-day and inter-day precisions at three spiked levels were 4.2%-16.2% and 6.9%-15.7%, respectively. This method was successfully applied to the determination of dechloranes in environmental water samples (laboratory tap water, reservoir water, wastewater treatment plant effluent, and landfill leachate treatment effluent). The recoveries of the three dechloranes at different spiked levels ranged from 77.8% to 113.3% with relative standard deviations (RSDs) of 2.5%-16.3% (*n*=3). With the advantages of operational simplicity, high sensitivity, and good reproducibility, the proposed method is suitable for the qualitative and quantitative determination of dechloranes in environmental water.

得克隆类物质是一类耐光、化学性质稳定的有机氯系脂肪族化合物,自溴代阻燃剂被禁止使用后,得克隆类物质被广泛应用于电子设备、纺织品等材料的加工生产中^[1]^。得克隆类物质极易通过磨损、溶解等方式释放到环境中^[2]^, 2006年Hoh等^[3]^首次在五大湖沉积物及鱼类中检出得克隆,随后得克隆在全球不同区域的环境和生物介质中被检出^[4]^。毒理学研究表明,得克隆具有神经行为毒性和生殖毒性,长期接触得克隆会对肺、肝脏等器官造成损伤^[5]^。2023年5月,得克隆被列入《斯德哥尔摩公约》持久性有机污染物清单中,同年中国也将其列入《重点管控新污染物清单》,并规定自2024年1月1日起,禁止得克隆的生产、加工使用及进出口。因此,迫切需要建立一种准确、高效、灵敏的分析方法,用于监测得克隆类物质在环境水体中的污染水平。

目前,气相色谱-负化学电离源质谱(GC-NCI/MS)是得克隆类物质的常用检测方法^[6,7]^。得克隆类物质在环境水体中以痕量水平(ng/L)存在,无法通过仪器对其进行直接检测,须先借助样品前处理技术进行富集浓缩,以满足仪器分析的要求。现阶段关于水中得克隆类物质的样品前处理技术主要有液液萃取(liquid-liquid extraction, LLE)^[8]^和固相萃取(solid-phase extraction, SPE)^[9]^。LLE的有机溶剂消耗量大且容易造成二次污染,而传统SPE技术易出现柱堵塞的问题,并且耗时较长。磁固相萃取(magnetic solid-phase extraction, MSPE)是一种新型样品前处理技术,具有操作简单、萃取时间短、样品用量小及环境友好等优势,在样品前处理领域得到了广泛的应用^[10,11]^。MSPE技术的核心在于磁性材料的选择,根据目标化合物的结构特性来制备磁性吸附材料有助于提高目标化合物的选择性。共价有机骨架(covalent organic frameworks, COFs)材料是由轻质元素组成、通过强共价键与有机单体连接而成的结晶性有机多孔材料^[12]^。COFs具有比表面积大、孔径可调、化学稳定性高及可功能化修饰等优点^[13]^,已被广泛应用于SPE领域^[14⇓-16]^。Zhang等^[17]^制备了一种氟化磁性COF(Fe_3_O_4_@TpPa-F_4_),并将其作为MSPE吸附剂用于牛奶中全氟化合物的富集。Guo等^[18]^将1,3,5-三(4-甲酰基苯基)苯-联苯胺(1,3,5-tris(4-formylphenyl)benzene-benzidine, TFPB-BD)键合到氨基化的不锈钢纤维上,并利用SPE技术实现了水产品中多氯联苯的分析检测。我们课题组^[19⇓-21]^开发了磁性COF材料和COF复合膜材料,并将他们用于环境水样中消毒副产物、杀菌剂及六溴环十二烷等化合物的样品前处理。

本文以通过原位合成法制备的磁性共价有机骨架材料(Fe_3_O_4_@TpBD)为MSPE吸附剂,结合GC-NCI/MS,建立了一种实际环境水体中3种得克隆类物质(得克隆603(Dec 603)、顺式得克隆(*syn*-DP)、反式得克隆(*anti*-DP))的分析检测方法,为环境水体中痕量得克隆类物质的灵敏筛检提供了技术支撑。

## 1 实验部分

### 1.1 仪器、试剂与材料

Trace1610 ISQ 7610气相色谱-质谱联用仪(美国Thermo Fisher Scientific公司); JEM-2100 F型场发射透射电子显微镜(日本JEOL公司); Lakeshore-7404型振动样品磁强计(美国Lakeshore公司); BRUKER D8 ADVANCE型X射线多晶衍射仪(德国Bruker AXS公司); Dataphysics-OCA20型接触角测量仪(德国Dataphysics公司); Frontier傅里叶变换红外光谱仪(美国PerkinElmer公司); Sigma 300型扫描电子显微镜(德国ZEISS公司); Millipore D-24UV超纯水机(美国Millipore公司)。

*syn*-DP(纯度99.5%)、*anti*-DP(纯度99.5%)标准品购自广州佳途科技股份有限公司;Dec 603标准品(50 μg/mL)购自美国Accustandard公司;2,4,6-三醛基间苯三酚(2,4,6-triformylphloroglucinal, Tp,纯度98%)购自上海梯希爱化成工业发展有限公司;联苯胺(benzidine, BD,纯度95%)和Fe_3_O_4_纳米粒子(20 nm,金属含量基数99.5%)购自上海阿拉丁化学试剂有限公司;丙酮(色谱级)购自国药集团化学试剂有限公司;正己烷(色谱级)购自上海麦克林生化科技有限公司;四氢呋喃(分析纯)购自天津市富宇精细化工有限公司。

实验用水均为超纯水机制备的超纯水(18.2 MΩ·cm)。实验室自来水采集于实验室,水库水采集于青岛市黄岛区,垃圾渗滤液处理水采集于青岛市垃圾填埋厂,污水处理厂出水采集于青岛市污水处理厂,4种水样用0.45 μm滤膜过滤至棕色玻璃瓶中,于4 ℃冰箱中保存。

### 1.2 Fe_3_O_4_@TpBD的制备

参照文献[19],采用原位合成法制备Fe_3_O_4_@TpBD。首先,将Fe_3_O_4_纳米粒子(96 mg)分散于22 mL四氢呋喃中,超声混匀30 min,加入48 mg BD,继续超声20 min,之后将上述混合体系转移至配有回流冷凝管的三颈圆底烧瓶中,于50 ℃下搅拌30 min;将24 mg Tp分散于8 mL四氢呋喃中,然后逐滴加入至上述混合体系中,在50 ℃下继续搅拌3 h;最后使用磁铁收集反应产物,即得Fe_3_O_4_@TpBD,分别使用2 mL四氢呋喃和2 mL甲醇涡旋洗涤Fe_3_O_4_@TpBD 3次,随后将Fe_3_O_4_@TpBD置于60 ℃真空干燥箱中干燥,取出后置于干燥器中,备用。

### 1.3 溶液的配制

分别准确称取*syn*-DP和*anti*-DP标准品5 mg(精确至0.01 mg),用正己烷溶解并定容至5 mL,得到质量浓度为1000 mg/L的标准储备液,置于棕色瓶中并在4 ℃冰箱中保存。分别准确移取50 μL *syn*-DP和*anti*-DP标准储备液及1 mL Dec 603标准品,用正己烷稀释并定容至5 mL,配制成质量浓度为10 mg/L的混合标准使用液,于4 ℃冰箱中保存,临用前再使用正己烷稀释至所需质量浓度。

### 1.4 样品前处理

称取20 mg Fe_3_O_4_@TpBD于50 mL离心管中,加入50 mL水样(pH 7),涡旋萃取10 min,之后利用磁铁进行分离,弃去上清液;向上述离心管中加入2 mL正己烷-丙酮(1∶1, v/v),涡旋洗脱6 min,之后进行磁分离并收集洗脱液,加入2 g无水硫酸钠,在30 ℃下氮吹至近干,用0.2 mL正己烷复溶,经0.22 μm滤膜过滤后,进行GC-NCI/MS分析。

### 1.5 分析条件

#### 1.5.1 色谱条件

色谱柱:TG-5SILMS石英毛细管柱(30 m×0.25 mm×0.25 μm);载气:氦气;载气流速:1.2 mL/min。色谱柱升温程序:初始温度80 ℃,保持2 min;以10 ℃/min升温至300 ℃,保持15 min。进样口温度:280 ℃;进样方式:不分流进样;进样量:1 μL。

#### 1.5.2 质谱条件

离子化方式:负化学电离源;离子源温度:200 ℃;传输线温度:280 ℃;反应气:甲烷;流速:1.25 mL/min;监测模式:选择离子监测(SIM)。3种得克隆类物质的保留时间及质谱参数等信息如表1所示。

**表1:** 3种得克隆类物质的保留时间和质谱参数

Analyte	Retention time/min	M_r_	Quantitative ion (m/z)	Qualitative ions (m/z)
Dechlorane 603 (Dec 603)	26.57	637.6	637.6	635.6, 639.6
syn-Dechlorane plus (syn-DP)	30.73	653.7	635.6	651.6, 655.6
anti-Dechlorane plus (anti-DP)	32.14	653.7	635.6	651.6, 655.6

### 1.6 吸附实验

#### 1.6.1 吸附动力学实验

由于3种得克隆类物质的化学结构及物理性质相似,本实验选取*syn*-DP作为吸附实验的考察对象。向50 mL实验室自来水中加入25 μL 1000 mg/L的*syn*-DP标准储备液,配制成初始质量浓度(*C*_0_)为500 μg/L的*syn*-DP待测溶液;向上述溶液中加入20 mg Fe_3_O_4_@TpBD,于25 ℃下进行涡旋处理,并在不同时间点(5、8、10、20、30、40、50、60 min)取样,每次取2 mL,用磁铁进行分离,随后测定溶液中*syn*-DP的质量浓度。根据式(1)计算Fe_3_O_4_@TpBD在不同时间点对s*yn*-DP的吸附量:


(1) $$q_{t}=\frac{\left(C_{0}-C_{t}\right)V}{m}$$

其中,*t*为吸附时间(min), *q_t_*为*t*时刻的吸附量(μg/g), *C_t_*为*t*时刻溶液中*syn*-DP的质量浓度(μg/L),*V*为吸附溶液初始体积(L), *m*为Fe_3_O_4_@TpBD的质量(g)。

#### 1.6.2 吸附等温线的测定

分别向50 mL实验室自来水中加入不同质量浓度的*syn*-DP标准储备液,配制成*C*_0_分别为10、50、100、500、1000 μg/L的*syn*-DP待测溶液;分别准确称取20 mg Fe_3_O_4_@TpBD,加入到上述溶液中,于25 ℃下涡旋处理10 min,随后用磁铁进行分离,测定上清液中*syn*-DP的质量浓度。

## 2 结果与讨论

### 2.1 Fe_3_O_4_@TpBD的表征

采用透射电子显微镜和扫描电子显微镜对Fe_3_O_4_@TpBD的形貌进行表征,结果如图1所示。在包覆了COF材料后,Fe_3_O_4_@TpBD形成了明显的核壳结构(图1a),该结果与文献[22]报道一致;由图1b和图1c可以看出,在包覆了COF材料后,Fe_3_O_4_@TpBD表面变得粗糙。上述结果说明,Fe_3_O_4_@TpBD已被成功制备。

**图1:**
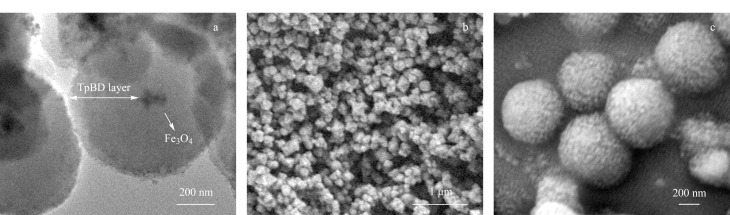
(a)Fe_3_O_4_@TpBD的透射电镜图;(b)Fe_3_O_4_和(c)Fe_3_O_4_@TpBD的扫描电镜图

图2为Fe_3_O_4_、TpBD和Fe_3_O_4_@TpBD的FT-IR图。591 cm^-1^处的特征吸收峰归属于Fe_3_O_4_的Fe-O-Fe^[23]^, 1452 cm^-1^和1572 cm^-1^处的两个特征吸收峰归属于TpBD中C=C键的伸缩振动^[24]^, 1288 cm^-1^和1621 cm^-1^处的两个特征吸收峰分别对应于TpBD中的C-N和C=O键的伸缩振动^[21]^。由图2可知,Fe_3_O_4_@TpBD同时包含了Fe_3_O_4_和TpBD的特征吸收峰,与文献[22]报道结果一致,证明了Fe_3_O_4_@TpBD的成功制备。

**图2:**
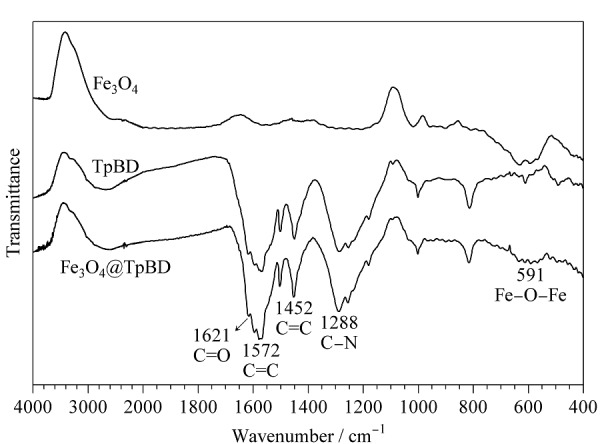
Fe_3_O_4_、TpBD和Fe_3_O_4_@TpBD的FT-IR图

利用XRD对Fe_3_O_4_@TpBD的晶体结构进行表征,结果如图3所示。与文献[23]报道结果一致,在30.2°、35.5°、43.1°、57.1°、62.6°处均出现了Fe_3_O_4_的特征衍射峰,分别对应于(220)、(311)、(400)、(511)、(440) 5个晶面;与文献[25]报道结果一致,在18.4°和26.4°处出现了TpBD的特征衍射峰,对应于(210)和(001)晶面。上述XRD结果均证明了Fe_3_O_4_@TpBD的成功制备。

**图3:**
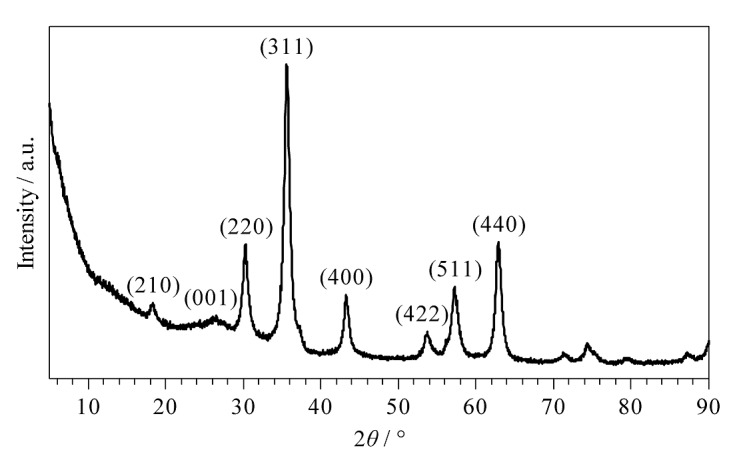
Fe_3_O_4_@TpBD的XRD图

利用振动样品磁强计测定Fe_3_O_4_@TpBD的磁滞曲线,结果如图4所示。Fe_3_O_4_@TpBD的饱和磁化值为23.7 emu/g,由文献[26]可知,该饱和磁化值足以保障外加磁场作用下的固液分离。由图4插图也可以看出,在外部磁铁作用下,Fe_3_O_4_@TpBD能够在15 s内实现快速聚集。上述结果表明,本文所制备的Fe_3_O_4_@TpBD具有强磁响应性,能够实现快速磁分离。

**图4:**
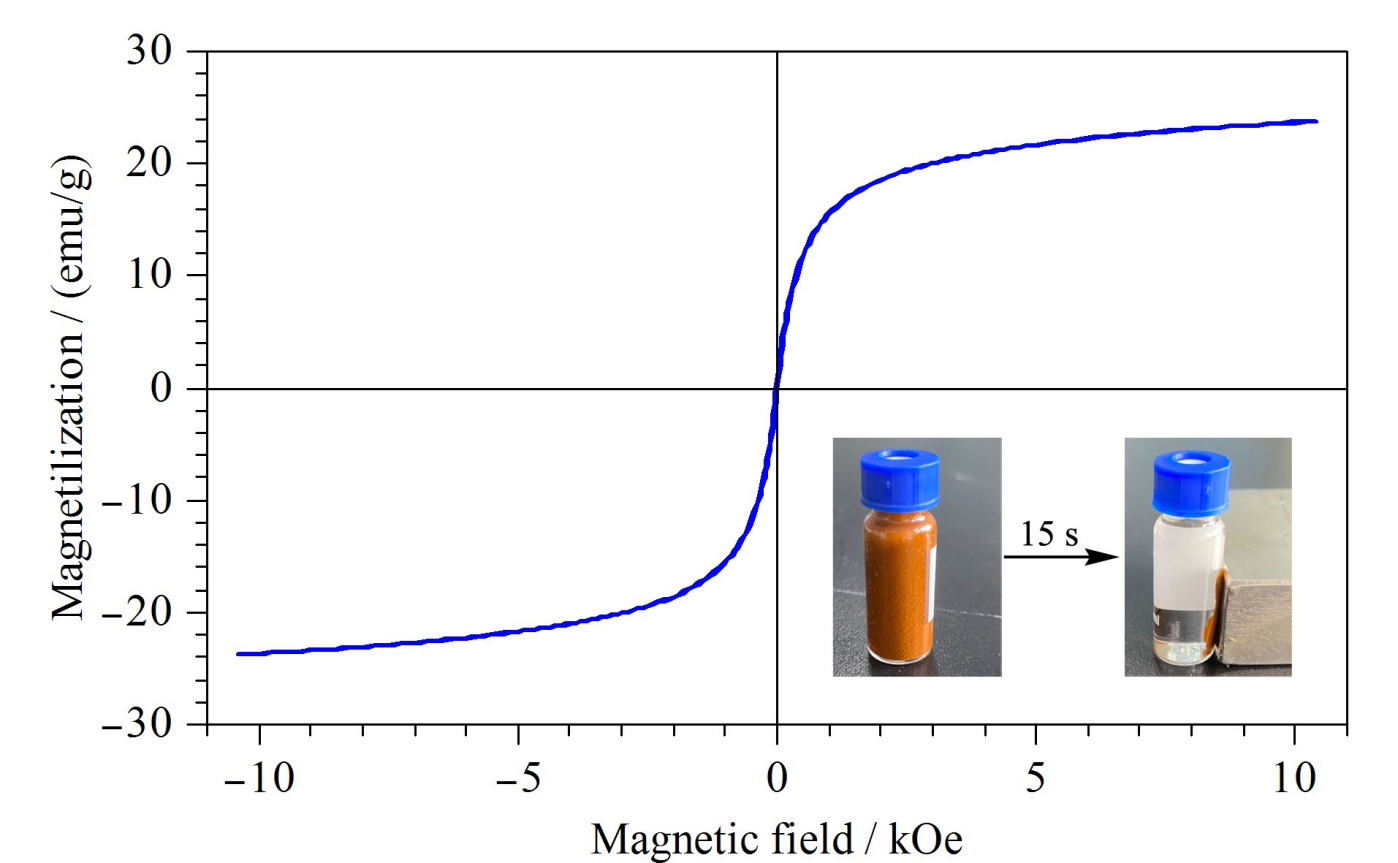
Fe_3_O_4_@TpBD的磁滞曲线

对Fe_3_O_4_@TpBD进行水接触角测试,以评估其疏水性,结果如图5所示。Fe_3_O_4_@TpBD的水接触角为84.1°,表明其具有较强的疏水性,可能是因为TpBD的结构中富含疏水性苯基。因此,本文所制备的Fe_3_O_4_@TpBD可为得克隆类物质的萃取提供疏水作用吸附位点。

**图5:**
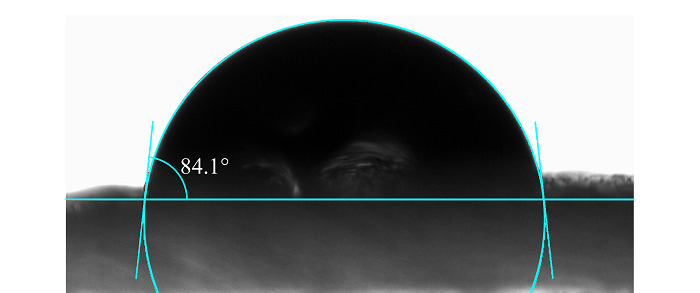
Fe_3_O_4_@TpBD的水接触角

### 2.2 吸附性能考察

#### 2.2.1 吸附动力学

吸附速率是评价吸附剂吸附性能的重要指标之一。Fe_3_O_4_@TpBD对s*yn*-DP的吸附动力学曲线如图6a所示,在吸附初期,*syn*-DP的吸附量随时间推移迅速增加,随后增加缓慢,在10 min时达到吸附平衡,经式(1)计算可得平衡吸附量(*q*_e_)为112.04 μg/g。本实验进一步利用准一级和准二级吸附动力学方程(式(2)和式(3))对*syn*-DP的吸附动力学过程进行分析:


(2) ln (*q*_e_-*q_t_*)=ln *q*_e_-*k*_1_*t*

(3) $$\frac{t}{q_{t}}=\frac{1}{k_{2}q_{e}^{2}}+\frac{1}{q_{e}}t$$

式中,*k*_1_和*k*_2_分别为准一级和准二级模型的吸附速率常数(0.026 min^-1^和0.0107 g/(μg·min))。

**图6:**
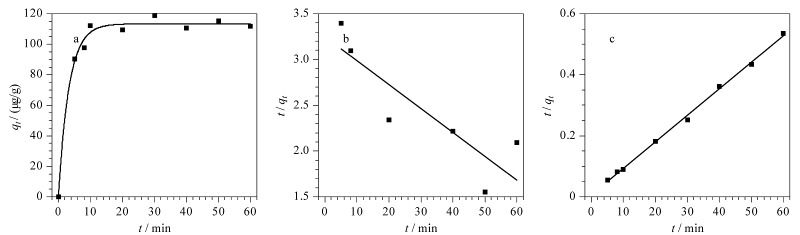
Fe_3_O_4_@TpBD对*syn*-DP的(a)吸附动力学曲线及(b)准一级、(c)准二级吸附动力学拟合曲线

吸附动力学拟合曲线如图6b和6c所示,由准一级吸附动力学模型拟合所得线性拟合相关系数(*R*^2^)为0.762,准二级动力学吸附模型的*R*^2^为0.997,后者大于前者,说明准二级吸附动力学模型可以更好地描述吸附动力学过程;同时上述结果表明,在Fe_3_O_4_@TpBD对*syn*-DP的吸附过程中,化学吸附起主导作用。对吸附原理进行推断,主要是基于Fe_3_O_4_@TpBD中大量的C=O(属于Lewis碱)和得克隆类物质分子中具有较强电负性的Cl原子(属于Lewis酸),二者之间可形成卤素键,同时Fe_3_O_4_@TpBD与得克隆类物质之间存在疏水作用力。

#### 2.2.2 吸附等温线

按照1.6.2节步骤进行吸附等温线的测定,采用Langmuir模型(式(4))和Freundlich模型(式(5))对吸附等温线进行拟合:


(4) $$\frac{C_{e}}{q_{e}}=\frac{1}{K_{L}q_{m}}+\frac{C_{e}}{q_{m}}$$

(5) $$\ln q_{e}=\ln K_{F}+\frac{1}{n}\ln C_{e}$$

式中,*C*_e_为*syn*-DP的吸附平衡质量浓度(μg/L), *q*_m_为最大理论吸附量(μg/g), *K*_L_为*syn*-DP的Langmuir吸附常数(0.0033 μg/L), *K*_F_和*n*分别为*syn*-DP的Freundlich模型吸附常数和强度系数。

Fe_3_O_4_@TpBD对s*yn*-DP的吸附等温线如图7a所示,随着*C*_e_的增加,*q*_e_也不断增加且增加趋势逐渐趋于平缓。两种模型的拟合结果如图7b和7c所示,通过Langmuir模型拟合所得*R*^2^为0.994,高于通过Freundlich模型拟合所得*R*^2^(0.971),说明Langmuir吸附模型更加贴切Fe_3_O_4_@TpBD对得克隆类物质的吸附过程。Langmuir模型假定吸附行为是发生在表面的单分子层,所有吸附点和吸附物之间具有相同的作用力。由实验结果推测,该吸附过程可能是单分子层吸附,吸附质分子间不存在相互作用力,当吸附位点被占满时,吸附过程达到饱和状态^[27]^。

**图7:**
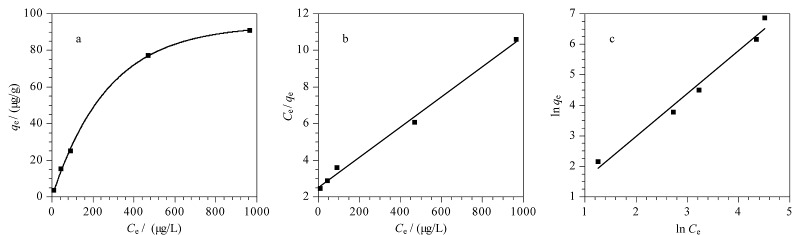
Fe_3_O_4_@TpBD对s*yn*-DP的(a)吸附等温线及(b)Langmuir模型和(c)Freundlich模型拟合结果

通过Langmuir模型计算可得,*q*_m_为120.48 μg/g,与实验所得*q*_e_ (112.04 μg/g)接近。吸附剂的吸附剂性能与Freundlich常数(1/*n*)有关,本实验中Fe_3_O_4_@TpBD对*syn*-DP的Freundlich常数为0.565,表明上述吸附过程容易进行^[28,29]^。

### 2.3 MSPE条件优化

为获得最佳萃取效果,采用单一变量法对MSPE过程中的Fe_3_O_4_@TpBD用量、水样pH、萃取时间、洗脱溶剂种类和体积、洗脱时间、离子强度等参数进行了优化。每组设置3个平行实验,水样中得克隆类物质的加标水平均为40 ng/L。

#### 2.3.1 Fe_3_O_4_@TpBD用量

在MSPE过程中,吸附剂的用量会直接影响萃取效果。如图8a所示,实验考察了Fe_3_O_4_@TpBD用量(5、10、15、20、25 mg)对3种目标分析物回收率的影响。随着Fe_3_O_4_@TpBD用量的增加,3种目标分析物的回收率逐步提高;当Fe_3_O_4_@TpBD用量为25 mg时,Dec 603的回收率继续上升,而*syn*-DP和*anti*-DP的回收率略有下降。因此最终选取Fe_3_O_4_@TpBD的用量为20 mg。

**图8:**
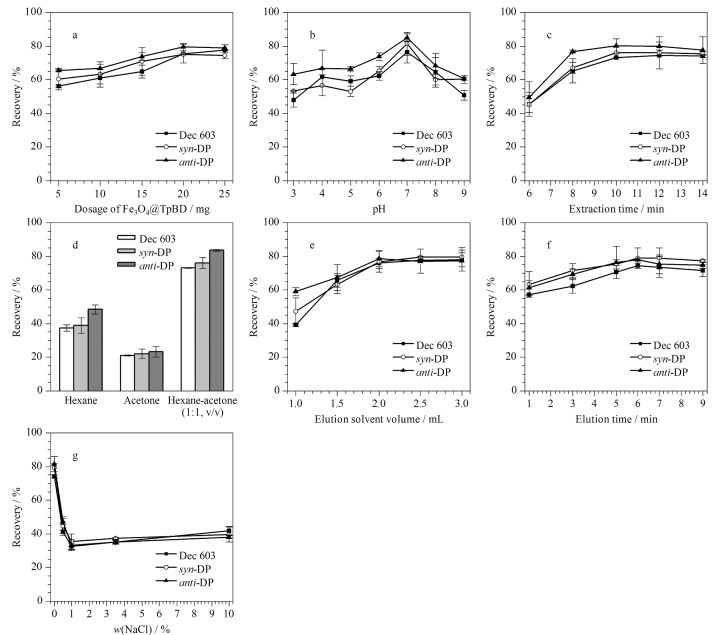
(a) Fe_3_O_4_@TpBD用量、(b)水样pH、(c)萃取时间、(d)洗脱溶剂种类、(e)洗脱溶剂体积、(f)洗脱时间、 (g)NaCl质量分数对3种目标分析物回收率的影响(*n*=3)

#### 2.3.2 水样pH

水样pH决定了Fe_3_O_4_@TpBD中官能团的存在形态,从而影响萃取效果。实验考察了不同水样pH(3~9)对3种目标分析物回收率的影响,其中水样pH通过0.1 mol/L盐酸和1 mol/L氢氧化钠溶液来调节。结果如图8b所示,当pH为7时,3种目标分析物的回收率最高。分析其原因,当水样pH<7时,水样中的H^+^与Fe_3_O_4_@TpBD中的C=O会形成配位共价键^[21,30]^,导致Fe_3_O_4_@TpBD的吸附位点减少,萃取效率降低;当水样pH>7时,水样中的OH^-^(路易斯碱)与目标分析物中亲电的Cl结合^[31,32]^,导致目标分析物的吸附量减少,萃取效率降低。因此最终确定水样pH为7。

#### 2.3.3 萃取时间

实验考察了不同萃取时间(6、8、10、12、14 min)对3种目标分析物萃取效果的影响,结果如图8c所示。当萃取时间从6 min增加到10 min时,3种目标分析物的回收率不断提高,继续增加萃取时间,3种目标分析物的回收率无明显变化,说明萃取时间为10 min时吸附过程已达到平衡。因此确定萃取时间为10 min。

#### 2.3.4 洗脱溶剂种类、体积及洗脱时间

在MSPE过程中,洗脱溶剂种类是影响回收率的关键因素。实验考察了正己烷、丙酮、正己烷-丙酮(1∶1, v/v)对3种目标分析物回收率的影响,其中洗脱体积均为2 mL,结果如图8d所示。实验结果表明,使用正己烷-丙酮(1∶1, v/v)洗脱时,3种目标分析物的回收率最高。随后对正己烷-丙酮(1∶1, v/v)的体积进行了优化,随着正己烷-丙酮(1∶1, v/v)体积的增加,3种目标分析物的回收率逐渐增大,当正己烷-丙酮(1∶1, v/v)体积超过2 mL时,3种目标分析物的回收率趋于稳定(图8e)。结合绿色化学原则,选择2 mL正己烷-丙酮(1∶1, v/v)用于后续实验。

对洗脱时间进行优化,当洗脱时间从1 min增加至6 min时,3种目标分析物的回收率达到最大,继续延长洗脱时间,3种目标分析物的回收率无明显变化或略有下降,结果如图8f所示。因此,最终选择洗脱时间为6 min。

#### 2.3.5 离子强度

实验考察了离子强度对3种目标分析物回收率的影响。向水样中加入不同质量分数(0、0.5%、1.0%、3.5%、10%)的NaCl,从图8g中可以看出,随着NaCl的质量分数从0增加至1%, 3种目标分析物的回收率持续降低,继续增大NaCl的质量分数,回收率无明显变化。造成上述现象的原因可能是过高的离子强度增加了溶液黏度,反而降低了目标分析物在溶液中的传质效率^[33]^。因此后续实验选择不加盐。最终,在上述优化的实验条件下,3种目标分析物均得到了良好的响应和分离,色谱图见图9。

**图9:**
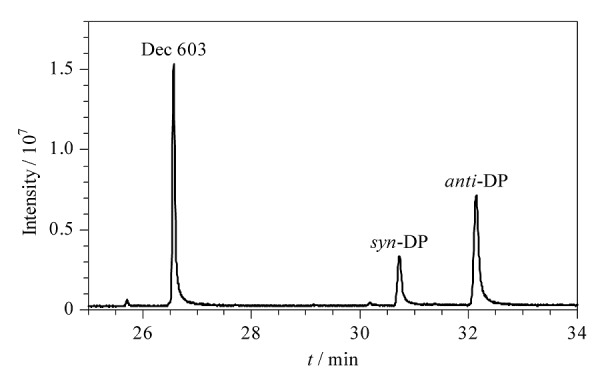
优化条件下3种目标分析物(1 mg/L)的色谱图

### 2.4 Fe_3_O_4_@TpBD的重复利用性

为考察Fe_3_O_4_@TpBD的重复利用性,以*syn*-DP和*anti*-DP为例,使用Fe_3_O_4_@TpBD对*syn*-DP和*anti*-DP进行5次吸附-洗脱循环。结果如图10所示,在重复循环5次后,*syn*-DP和*anti*-DP的回收率无明显下降,表明Fe_3_O_4_@TpBD具有较好的重复利用性。

**图10:**
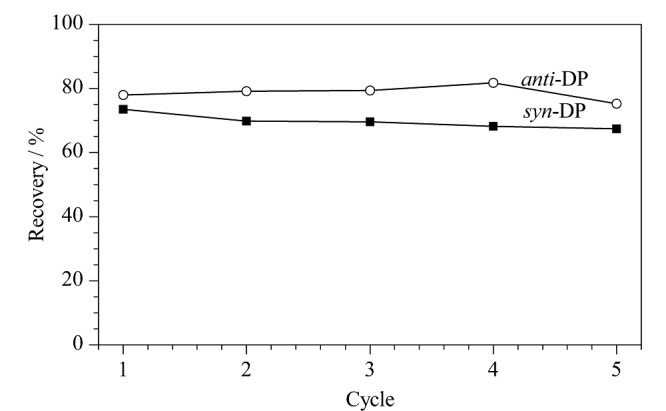
Fe_3_O_4_@TpBD的重复利用性(5次吸附-洗脱循环)

### 2.5 基质效应

选取实验室自来水、水库水、污水处理厂出水和垃圾渗滤液处理水4种基质样品来考察基质效应(ME)。按照1.4节步骤对4种基质样品进行前处理,获得空白基质液,再用4种空白基质液配制系列质量浓度(2、8、20、50、100、400、1000 ng/L)的基质匹配混合标准溶液,同时用超纯水对3种标准品进行逐级稀释,配制成相同质量浓度的溶剂混合标准溶液。对上述两种溶液进行样品前处理后,进样分析。按公式ME=(基质匹配混合标准曲线斜率/溶剂混合标准曲线斜率-1)×100%来计算ME。|ME|<20%为弱基质效应,可忽略且无需采取补偿措施;20%≤|ME|≤50%为中等程度基质效应,需采取补偿措施;|ME|>50%为强基质效应,需采取补偿措施^[34]^。实验结果表明,在4种基质样品中,3种目标分析物的ME分别为13.7%~29.7%、41.3%~62.8%、44.4%~58.5%和13.2%~15.3%,存在较强的基质效应。因此,为有效消除基质效应所带来的干扰^[35]^,本研究采用基质匹配混合标准溶液进行后续定量分析。

### 2.6 方法学考察

#### 2.6.1 线性范围、检出限和定量限

用4种空白基质液将3种目标分析物的混合标准使用液稀释成系列质量浓度(2、8、20、50、100、400、1000 ng/L)的模拟水样,进行样品前处理后上机检测。以目标分析物的质量浓度为横坐标(*x*, ng/L)、峰面积为纵坐标(*y*),绘制工作曲线。结果表明,3种目标分析物在2~1000 ng/L范围内线性关系良好,以信噪比(*S/N*)分别为3和10时所对应的质量浓度作为检出限(LOD)和定量限(LOQ),3种目标分析物的LOD为0.18~0.27 ng/L, LOQ为0.60~0.92 ng/L,以实验室自来水基质为例,相关数据见表2。

**表2:** 3种目标分析物的线性范围、相关系数、检出限及定量限

Analyte	Linear range/(ng/L)	Linear equation	r^2^	LOD/(ng/L)	LOQ/(ng/L)
Dec 603	2-1000	y=19.94x-86.59	0.9974	0.18	0.60
syn-DP	2-1000	y=1.96x+60.18	0.9997	0.22	0.73
anti-DP	2-1000	y=7.71x-54.27	0.9978	0.27	0.92

*y*: peak area; *x*: mass concentration, ng/L.

#### 2.6.2 回收率和精密度

在实验室自来水样品中添加低、中、高3个水平(8、50、200 ng/L)的得克隆类物质混合标准使用液,按照上述前处理方法进行加标回收试验,计算回收率;每个加标水平测定6次,计算日内精密度;连续测定6天,计算日间精密度。结果如表3所示,在3个加标水平下,3种目标分析物的回收率为79.8%~108.0%,日内和日间精密度分别为4.2%~16.2%和6.9%~15.7%。此外,以加标40 ng/L的实验室自来水样品为例,其经MSPE富集前后的色谱图如图11所示,结果表明,3种得克隆类物质均获得了有效富集。上述实验结果说明,该方法的重复性和准确性良好,适用于环境水体中得克隆类物质的分析检测。

**表3:** 3种得克隆类物质的回收率和日内、日间精密度(*n*=6)

Analyte	Spiked level/ (ng/L)	Recovery/ %	Intra-day RSD/%	Inter-day RSD/%
Dec 603	8	108.0	4.2	9.4
	50	106.3	16.2	15.7
	200	97.6	14.9	14.5
syn-DP	8	96.4	5.5	9.0
	50	86.6	15.2	6.9
	200	81.0	9.6	9.2
anti-DP	8	98.5	10.8	8.7
	50	97.9	15.1	12.0
	200	79.8	11.5	7.2

**图11:**
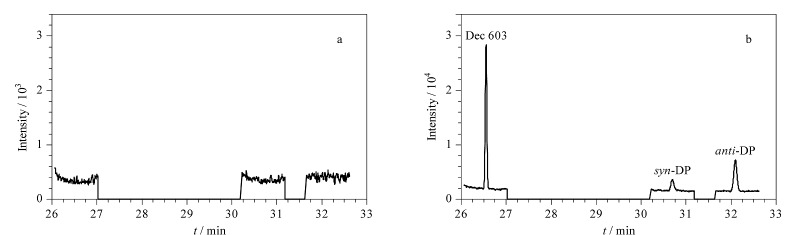
实验室自来水样品(加标水平40 ng/L)经MSPE富集(a)前、(b)后的色谱图

### 2.7 方法应用

在最佳实验条件下,对实验室自来水、水库水、污水处理厂出水和垃圾渗滤液处理水4种环境水样中的得克隆类物质进行检测,以验证所建方法的准确性。实验结果表明,4种环境水样中均未检出3种得克隆类物质。在低、中、高3个加标水平(8、50、200 ng/L)下分别对水库水、污水处理厂出水和垃圾渗滤液处理水3种环境水样进行加标回收试验,结果如附表1(www.chrom-China)所示,3种目标分析物的回收率为77.8%~113.4%,相对标准偏差为2.5%~16.3%。

### 2.8 与文献方法的比较

将本文所建方法与文献报道方法进行比较。结果如表4所示,与其他文献方法相比,本方法的样品前处理时间较短,检出限较低,基质样品种类较多,有机试剂用量更少,能够满足环境水体中得克隆类物质的测定需求。

**表4:** 本方法与文献方法的比较

Analysis method		Pretreatment method		Matrices	Analytes	Pretreatment time	Organic reagent dosage/mL	LODs/ (ng/L)		Ref.
GC-NCI/MS	LLE		seawater		Dec 602, Dec 603, Dec 604, syn-DP, anti-DP	>10 min	200	0.01-	0.1	[36]
GC-NCI/MS	LLE		aquaculture water		syn-DP, anti-DP	1 h	300	52-	66	[37]
GC-ECNI/MS	SPE		drinking water		Dec 602, Dec 603, syn-DP, anti-DP	>1 h	>30	0.19-	0.44	[38]
GC-EI/MS	vortex		adhesives		syn-DP, anti-DP	10 min	30	43-	56	[39]
GC-NCI/MS	MSPE		laboratory tap water, reservoir water, wastewater treatment plant effluent, landfill leachate treatment effluent		Dec 603, syn-DP, anti-DP	16 min	2	0.18-	0.27	this work

NCI: negative chemistry ionization; ECNI: electron capture negative ionization; EI: electron impact; LLE: liquid-liquid extraction; SPE: solid-phase extraction.

## 3 结论

本研究制备了磁性共价有机骨架材料Fe_3_O_4_@TpBD,并将其作为MSPE吸附剂,建立了环境水体中3种得克隆类物质的GC-NCI/MS分析方法。所制备的Fe_3_O_4_@TpBD重复利用性高,磁响应性强且磁分离速度快。所建方法操作简单、快速,灵敏度高,样品前处理时间短,可为环境水样中得克隆类物质的残留分析提供技术支持。
